# The slippery difficulty of ever containing drug resistance with current practices

**DOI:** 10.1007/s10096-016-2855-x

**Published:** 2016-11-28

**Authors:** R. Fullybright

**Affiliations:** Department of Applied Research, Applied-Research Center for True Development, 4016 rue Prefontaine, Montréal, Québec H1W 0A3 Canada

**Keywords:** Antibiotic, Drug, Resistance, Rate, Pathogen, Pharmaceutical, Infectious, Disease

## Abstract

It has previously been shown that the rate of drug resistance emergence in medicine is exponential, while we have been producing drugs at a much lower rate. Our ability to successfully contain resistance at any one time is function of how many drugs we have at our disposal to counter new resistances from pathogens. Here, we assess our level of preparedness through a mathematical comparison of the drug manufacture rate by the pharmaceutical industry with the resistance emergence rate in pathogens. To that effect, changes in the rates of growth of the drugs production and resistance emergence processes are computed over multiple time segments and compared. It is found that new resistance emergence rate in infectious diseases medicine remains mathematically and *permanently* ahead of the drugs production rate by the pharmaceutical industry. Consequently, we are not in a position to ever contain current or future strengths of resistance from pathogens. A review of current practices is called for.

## Background

Once the rate of new resistance emergence in infectious diseases medicine is known and once the rate of drugs production by the pharmaceutical industry is also known, we can estimate our level of preparedness in the face of drug resistance from pathogens. Based on previously-established statistical models, we can assess whether we are currently overwhelmed by pathogen resistance or whether we stand to successfully contain it. How well we will be faring in the near or distant future can also be predicted. Analysis of the rates gap reveals that we are currently running behind pathogen resistance and that we also have no chance of overcoming it in the future should we continue with our current ways—unless required changes are made.

## Introduction

Drug resistance has been known to occur to essentially all drugs designed to target pathogens and, while antibiotics production by the pharmaceutical industry has come to almost a halt over the past 30 years [1], renewed efforts are now being made to jump-start or even accelerate [2] new antibiotics production to replace old ones rendered ineffective by pathogens. Therefore, it matters that we be aware of how fast our sustained rate of drugs production needs to be in order for us to not fall into another spell of antibiotics dryness and stay constantly ahead of the game.

Within that framework, we already know that drug resistance emergence rate, based on pathogen resistance data collected from a 90-year time period, involving 90 infectious diseases, 118 pathogens, and covering 337 molecules, is exponential [3]. And our approach to overcoming emerged resistance is to manufacture and introduce new drugs to pathogens. So, in order to remain prepared and effectively keep drug resistance under control, we need to be producing drugs at a rate greater than or at least equal to the rate of new resistance generation by pathogens. But the question is: Can we sustain an exponential rate of drugs production over the long-term? —Here and now, we assess our capacity to successfully contain resistance with a sustainable rate of antibiotics production, based on our current approach to the management of infectious diseases.

## Methods

Using the trends shown by predictive statistical models of drug resistance emergence in infectious diseases medicine, we estimate our present and future preparedness to effectively contain resistance and proceed to show how difficult it is going to be for the medical community to bring resistance under control. Evaluation of our level of preparedness is based on mathematical rates comparison of the resistance emergence process in pathogens with the drugs production process by the pharmaceutical industry. *Antibiotic*, *drug*, or *molecule* as used in this discussion covers all pathogen-killing agents.

## Results and discussion

Drug resistance emergence in pathogens has been shown to occur in layers, each layer being characterized by its own rate of resistance emergence [3]. The predictive statistical model which was found to characterize the rate of cumulative first-layer (i.e., monotherapy) resistance build-up across infectious diseases medicine, with molecules as a predictor, was [3]:

1 $$ {\mathrm{S}}_1=10.90{\textstyle\ \hbox{--}\ }0.417\mathrm{m}+0.01{\mathrm{m}}^2 $$

where S_1_ is the cumulative monotherapy resistance and *m* the cumulative number of molecules introduced to pathogens.

However, the predictive statistical model which was found to characterize the rate of cumulative monotherapy (single molecules) drugs manufactured by the pharmaceutical industry, with time as a predictor, was [3]:

2 $$ \mathrm{m}=-2.312{\textstyle\ \hbox{--}\ }0.428\mathrm{t}+0.056{\mathrm{t}}^2 $$

where *m* is the cumulative number of molecules introduced to pathogens and *t* elapsed time (years), with 1922 marking year 1.

For all practical purposes, we assume that the trend characterizing both processes (the drugs production and the resistance emergence processes) remains the same into the future.

Having the rates of both processes presented as in Eqs. 1 and 2, let us take a look at what happens in the future between the number of molecules available to control pathogens and the number of resistances the pathogens would have generated.

### Drug resistance in the distant future

Here, we shall work with time as a predictor.

For a distant time into the future, *t* heads to infinity. For such a distant time, the number of resistant combinations generated by pathogens and the number of molecules available to circumvent them would, each, be given by the mathematical limit of the functions represented by Eqs. 1 and 2 respectively. In both cases, the limit is calculated to be infinity. However, as we consider only the time variable and substitute *t* for *m* in Eq. 1, the cumulative resistance S_1_ becomes a fourth-degree polynomial with *t* as a variable (Table 1). Because both processes (resistance emergence and drugs production) increase to infinite levels in the distant future, the prevailing process in the future will be indicated by the limit of the ratio of both functions. However,

$$ \begin{array}{c}\hfill \mathrm{f}\mathrm{o}\mathrm{r}\kern0.5em \mathrm{all}\kern0.5em t\kern0.5em \epsilon \kern0.5em \mathrm{R},\hfill \\ {}\hfill \underset{+\infty }{ \lim \left({\mathrm{S}}_1/m\right)}=\underset{t\to +\infty }{ \lim \left({t}^2\right)}=+\infty \hfill \end{array} $$

So, as time goes by, the first-layer resistance-strengthening process in pathogens will stay ahead of our drugs production process. And this only relates to the 1st layer, which we cannot keep pace with, much less the 2nd, 3rd, 4th, … layers. As a result, there is not much hope for a successful control of drug resistance in the distant future.

**Table 1:** Table 1: Cumulative monotherapy drug resistance function

*Cumulative monotherapy drug resistance as an exponential or polynomial function*						
New, yearly drug resistance emergence across infectious diseases medicine was modeled, and was shown to grow as an exponential function [3]. But for purposes of the analysis made here, what we need is cumulative resistance, which is the summation of the new resistances in all previous years. Because new resistance in each year is exponential, without even modeling cumulative resistance per se, the latter can be expressed as the sum of exponentials and is therefore also an exponential function.						
However, although cumulative resistance grows exponentially, it can also be expressed as a polynomial function (as in Eq. 1) because the Taylor series expansion of a function, including exponential functions, allows the said function to be approximated by a sum of polynomials. So, the exponential cumulative resistance can also be expressed as a polynomial function. In fact, we have systematically modeled cumulative monotherapy resistance [3] and have found that the best model to characterize it is a 2nd-degree polynomial as shown in Eq. 1, using *m* as a variable (or a 4th-degree polynomial, using *t* as a variable, upon substitution).						
To further convince that cumulative monotherapy resistance can accurately be expressed either as an exponential function or a polynomial function, a graph of each of those functions is presented, based on real data (cf. Fig. 1). It can be seen in Fig. 1 that the exponential and polynomial graphs are essentially identical to each other, showing that cumulative resistance can be expressed as either one of those two functions.

**Fig. 1:**
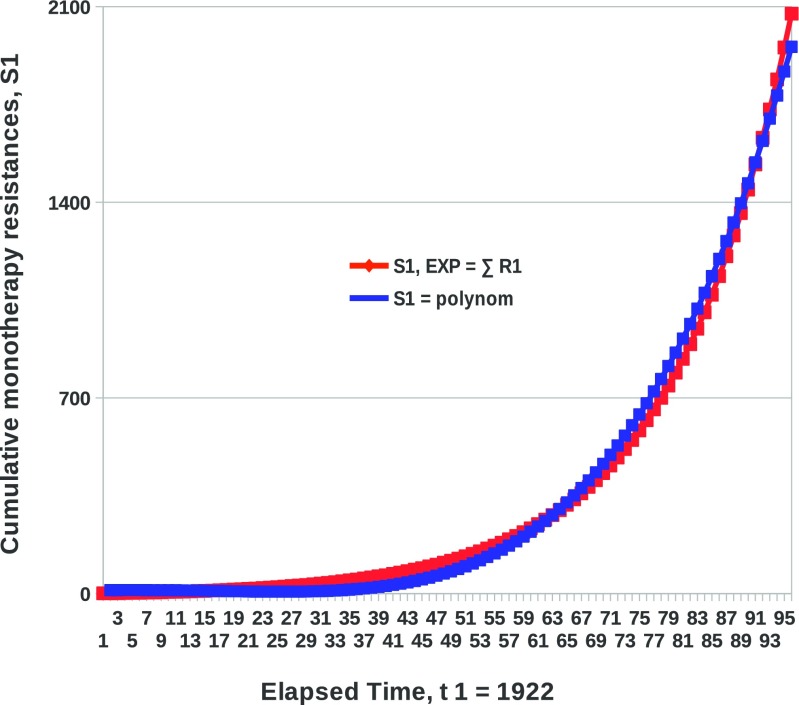
Cumulative monotherapy drug resistance in medicine—expressed either as a polynomial or as an exponential function. The *red graph* is the continued sums of the yearly, exponential, new monotherapy resistances, R_1_ [3]. As such, the red graph, representing the cumulative monotherapy resistance, is also exponential. The *blue graph*, however, is the graph of the 4th-degree polynomial function obtained through systematic modeling of true observations of cumulative monotherapy resistance data. The nearly identical shape of the two graphs shows that cumulative monotherapy resistance in medicine can be expressed either as a 4th-degree polynomial function or as an exponential function

### Drug resistance at the present time

Typically, a new molecule is produced in order to replace an earlier molecule which has become ineffective due to resistance. However, the earlier molecule together with its target pathogen made up one resistant pathogen–molecule combination [3]. The new molecule, together with that same target pathogen, makes up another pathogen–molecule combination which is not yet resistant and is meant to replace the previous *resistant* pathogen–molecule combination. Generally, every new molecule introduced to pathogens by the pharmaceutical industry is bound to enter a combination with a target pathogen, and this new combination is meant to be a replacement for a previous resistant combination.

Using Eq. 2 above, the cumulative number of molecules produced by the pharmaceutical industry in 2014 (*t* = 93) is expected to be 442 molecules. However, using Eq. 1, the first-layer cumulative resistance generated by pathogens when 442 molecules have been introduced to them is S_1_ = 1,782 resistances (1,782 resistant combinations).

From 2014, let us step ahead to 2015 and see what is happening:

Using Eq. 2, the cumulative number of molecules produced in 2015 (*t* = 94) is about 452, meaning that ten new additional molecules would have been introduced (from 2014 to 2015) to replace ten combinations which would have become resistant. Using Eq. 1, however, the first-layer cumulative resistance generated by the pathogens when 452 molecules have been introduced to them is S_1_ = 1,868 resistances—which is 86 resistant combinations more than in 2014.

So, we would be facing 86 new resistant combinations in 2015 while we would have introduced just ten more molecules which can replace only ten resistant combinations. We have 10 < 86. The difference is 86–10 = 76. We are going bankrupt.

That result means that the pathogens develop resistance to more molecules than have been introduced to them: to the ten molecules introduced (from 2014 to 2015) *and* to a difference of 86–10 = 76 *theoretical* or *abstract* molecules which Research & Development might *not have created yet*. That is it for the 1-year time slot from 2014 to 2015. We have seen that the pathogens are bound to stay ahead of us in the distant future; however, this shows that they are already ahead of us even at the present time.

Knowing that cumulative resistance build-up is characterized by a function (Eq. 1) whose first-order derivative remains dependent on the variable (*t* or *m*), it can be stated that the gap will keep widening anyway. Therefore, we should expect to lag farther and farther behind pathogens and to mathematically *never* be able to control resistance. This expectation can be verified with similar calculations for another one-year time slot; for example, from 2019 to 2020.

The calculations reveal a similar but worse trend:

Using Eq. 2, the cumulative number of molecules manufactured by the pharmaceutical industry in 2019 (*t* = 98) is expected to be about 494. However, using Eq. 1, the first-layer cumulative resistance generated by the pathogens when 494 molecules have been introduced to them is S_1_ = 2,241 resistances.

In 2020 (*t* = 99), the cumulative number of molecules manufactured is expected to be about 504, meaning that ten new additional molecules would have been introduced to replace ten combinations which would have become resistant. Using Eq. 1, the first-layer cumulative resistance when 504 molecules have been introduced to the pathogens is S_1_ = 2,343 resistances, which is 102 resistant combinations more than in 2019.

So, we would be registering 102 additional resistant combinations in 2020 while we would have introduced just ten newer molecules to the pathogens (from 2019 to 2020) to replace ten previously resistant combinations. We have 10 < 102. The difference is 102–10 = 92, which is even greater than the difference of 76 obtained for time slot 2014 to 2015. So, we are going even more bankrupt.

An apparent underlying characteristic of the drug resistance phenomenon is, therefore, that the pathogens develop resistance to *more* molecules than have been introduced to them. In this latest example for time slot 2019 to 2020, the pathogens develop resistance to the ten molecules introduced *and* to 102–10 = 92 *theoretical* or *abstract* molecules which Research & Development might *not have created yet*.

For the 2014–2015 time slot, we were bankrupt by 76 outstanding resistances that we were unable to control. However, for the 2019–2020 time slot, we became even *more* bankrupt by 92 outstanding resistances that we cannot control. As a result, we will try to control those resistances by manufacturing even more molecules, and the pathogens will respond to these newer molecules by developing an even greater number of resistances. Then, we will try to control each of those newer resistances by manufacturing even more molecules, and then the pathogens will respond to these newer molecules by developing an even greater number of resistances; so forth. This is a dangerous game which will quickly spiral out of control—and *is already doing so*. The picture arising out of this is that *the more molecules* we introduce to pathogens, the *more dangerous* the situation becomes. With every new molecule we manufacture and put on the market to control pathogens, resistance *gets worse*. So, molecules act exactly like wood feeding the fire that drug resistance constitutes.

Although we may be tempted to think that this situation characterizes only the first resistance layer (monotherapy drugs), the situation remains the same across-the-board, for all the layers of resistance (i.e., for dual-therapy drugs, triple-therapy drugs, etc.).

On the other hand, knowing that new drug resistance emergence rate in medicine is exponential and that the drug manufacture rate by the pharmaceutical industry, for all practical purposes, is linear, or, at best, a second-degree polynomial [3], it could have been seen, without even getting into the above computations, that, from a mathematical perspective, because an exponential function grows faster than and stays permanently ahead of a linear one, we will *never be able* to control resistance with our current approach, however disappointing this may be.

However, even if, by some stroke of luck, we were able to generate drugs at an exponential rate, with coefficients greater than those of the exponential rate of resistance emergence, the issue will still remain because molecules nurture resistance development in pathogens, and exponentially so— as discussed.

## Implications

Among other things, this result means that the pathogens become resistant to molecules ahead of us, ahead of time, and in an *abstract* manner. That is, the potential to be resistant to a molecule develops in the pathogen although that molecule might *not* yet be created by R&D. This is dangerous. Why? —Because the capacity develops in the pathogen but remains *latent*, *unmanifest*. When 10 to 15 years later, we eventually conceive and manufacture one of those 76 or 92 (theoretically) already-resisted molecules and discover that the pathogen is resistant to it, we would probably call that a case of *natural* or *intrinsic* resistance [4]. However, it is what we could rather call *entrained resistance*—because we would have “trained” the pathogens to become resistant to that molecule years earlier. This phenomenon partly explains the observation that some pathogens display resistance to molecules they haven’t even been exposed to [5], which means that the pathogen has developed resistance to the molecule prior to having encountered that molecule in actuality. *Entrained resistance* favors such an occurrence. Due to the high (exponential) rate of resistance emergence in just the first and second layers of resistance, *entrained resistance* is an important feature we need to begin considering.

The situation is devastating not just because of the high resistance emergence rate but precisely because the very *solution* that we have been applying makes the situation *even worse*. In fact, the only solution we have had has been to manufacture new molecules in order to circumvent resistance developed to previous molecules. However, from the above discussion, it can be seen that the more molecules we manufacture and introduce to pathogens, the more dangerous we make the situation become. Practically, we are involved in a competition with pathogens and do not stand to win that competition if something does not change radically in our resistance control strategies. Nevertheless, for those strategies to change, the understanding underpinning them will also need to undergo change. Thorough rectification is needed.

What this means is that if we continue to manufacture and introduce newer and newer molecules to pathogens, we can expect that the situation will get worse and worse. The competition will be lost regardless of any perspective which may encourage us to maintain our current practices. For example, one such perspective currently advocated for resistance control is to reduce antibiotics use. However, it can be seen from the foregoing that campaigning for a reduction in antibiotics use will *not* stop new resistance emergence but will only *delay* it (in time). And then, when it eventually emerges, the increase in new resistances will be exponential—with entrained resistance right behind it. So, the gap will continue to grow, and grow abysmal.

In other words, this means that if we recognize the danger posed by molecule-induced resistance and begin working towards overcoming it, then the solution can no longer be to produce even more molecules or to re-use old molecules in novel combinations, which is what we are currently doing. Such a proposition will hurt and can only hurt because molecules *induce resistance* in pathogens, and, resistance emergence is an *exponential* function of the number of molecules introduced to the pathogens [3]. So, extreme care is needed here because the above means that *the more molecules we introduce to pathogens, the worse we make their resistance become*.

In fact, what’s happening is something like this: we begin by introducing one molecule to the pathogens’ system, and, because resistance emergence rate as a function of molecules is exponential, they return more than one, let’s say five, new resistances to us. Then, we manufacture five new molecules and introduce these to the pathogens in order to control or circumvent those five new resistances, and get, say, 11 new resistances back. Then, we manufacture 11 new molecules and introduce them to the pathogens in order to control or circumvent those 11 new resistances, and get, say, 25 new resistances back. Then, we manufacture 25 new molecules and introduce them to the pathogens in order to control or circumvent those 25 new resistances, and get, say, 40 new resistances back. So on.

*How do you control a process like that?* —A nightmare which keeps running out of control.

As a striking, real-life example, it was found that, as of 2007, the medical community had introduced 337 molecules to the pathogens and that this has generated 1,163 cases of mono-therapy resistance [3]. So, we’ve gotten roughly three times more resistances than the number of molecules we have introduced to the pathogens. Let us now assume that we manufacture and introduce 1,163 new molecules to the pathogens in order to control/avoid/circumvent those 1,163 resistances. Assuming the same three-fold rate, we shall be getting roughly 1,163 × 3 ≈ 3,300 new cases of resistance. It keeps on expanding. How do you *contain* something that only *keeps expanding? *It is not containable.

It can be seen that molecules feed resistance and introducing more molecules to the pathogens causes resistance to worsen. So, our resistance-control approach, as it stands right now, is *not* sustainable. As we move forward with our current understanding and practices, attempting to control and contain resistance through a variety of proposed strategies, the realization needs to occur that resistance can *neither be contained nor controlled*. This is because of the second law of resistance, which states that resistance can only increase to infinite levels if not fully suppressed [6]. Indeed, if each new molecule we introduce to pathogens generates a new resistance, AND the only solution we have had has been to manufacture a new molecule and introduce it to pathogens (in order to bypass a pre-existing resistance), then cumulative resistance *can only increase infinitely*. This is only logical. Here, infinite resistance means an infinite number of resisted molecules—because resisted molecules are what we are counting. However, we still get infinite resistance even as this resistance is expressed not in terms of resisted molecules but in units of resistance [7]. Therefore, we can *never* contain resistance because the system is heading to infinity and infinity can *not* be contained. *No* advocated containment strategy can work (and *none* has been working!) because the system can *only* expand to infinity. This is because of the second law. That no containment strategy has been working is shown by the fact that the crisis has only been worsening, as reported by a number of sources [e.g., 8]. Hopefully, the suggestion made pertaining to needed changes in drug design [6] will bring the dilemma to a close and finally give the medical community the upper hand.

As a result of the foregoing, to talk about resistance containment or control is a mistake—because it is *impossible* to. Resistance can neither be contained nor controlled because it continues to *flare up every time a new molecule is introduced to the pathogens*. The immediate implication is that every effort now being made [8–10] and geared towards the production of newer and newer antibiotics (as we currently understand them) needs to immediately stop. And the sooner the better, although this may sound hard to digest.

The fact that antibiotic molecules act like wood feeding the fire that drug resistance constitutes may be counterintuitive but that’s what’s happening. So, any proposition encouraging the pharmaceutical industry to produce even more antibiotics (as we currently understand them), supposedly to help us deal with pathogen resistance, is dangerous and *effectively corners us even further*. It can be expected that the proposition according to which we will successfully control drug resistance by jump-starting antibiotics production will not materialize and cannot materialize. Such a proposition puts populations at greater and greater risks because of the exponential rise in resistance that new molecules induce. However, the problem would remain even if the rate were not exponential but were any other strictly-increasing mathematical function.

The school of thought on resistance control so far has been to speed up the discovery and production of newer antibiotics together with a reduction in antibiotics use [10–13]. Consequently, while our rate of new molecules production has not been high, we have been making increased use of combinations of old antibiotics, which also feeds resistance. Furthermore, even as efforts are now being made worldwide to phase out the routine use of antibiotics from animal production and save them only for human use [12], the problem remains and will continue to persist because the simple fact of *using* antibiotics, in humans or animals, is what causes the situation to get worse; the same way gasoline poured on fire causes the fire to rage even more. In fact, we have been thinking that it’s the *amount used* [14, 15]; but it’s not just the amount used. It is also the *simple fact of using*; what matters being the *essence* of the molecule. We need to come to grips with this.

The direness of the situation is not only due to *how much* antibiotics we are using but also to the simple fact of *using* antibiotics (as we currently understand them). This is because new resistance emergence and cumulative resistance build-up are, each, both quantity-dependent *and* molecules-dependent. In fact, in Eq. 1 and in others [3], molecules alone were found to be an excellent predictor of cumulative resistance build-up, although *no* explanatory variable for *quantity* (or mass) was present in the models. On the contrary, it is now argued that *abuse* (meaning *quantity*) of antibiotics is the culprit [14–17], and the molecules component is totally ignored. However, the *quantity* (say, kilograms) of antibiotics we use is a culprit but not the sole culprit because cumulative resistance build-up, after being quantity-dependent (it is dependent on new resistance emergence, and new resistance emergence is quantity-dependent), is strongly molecules-dependent. *Quantity*, when it is high (i.e., high drug pressure), only acts to shorten the duration required for the pathogen to develop resistance to the molecule. But then, once that resistance has emerged, the multiplicity of additional molecules to which the pathogen simultaneously displays resistance (say, as a result of cross-resistance), leading to an increase in the cumulative number of resisted molecules, is *not* affected by the quantity of the molecule used but *by the simple fact of having used that molecule*. The *essence* of the molecule (or drug) matters. 

Let us consider an example to illustrate:

Molecules *a* and *b* are of the same class and only molecule *a* currently exists, *b* is not yet manufactured. We’ve been treating the pathogen with molecule *a* to the point that resistance develops to it. True, resistance has developed to molecule *a* because of drug pressure (a given quantity of molecule *a* has been applied to the pathogen over time). However, a few years later, we manufacture molecule *b*, introduce it to the pathogen, and discover that the pathogen is resistant to it (a case of *entrained resistance*). *Question:* Has the pathogen become resistant to molecule *b* because of drug pressure? *Answer: No*, because no quantity of molecule *b* has been applied to the pathogen (yet the pathogen has displayed resistance to the molecule). Note needs to be taken that such cross-resistance can also be conferred to a molecule of a totally different class [18]. Therefore, *quantity* is *not* involved in the spread of pathogen resistance to multiple molecules. *Quantity* is indeed involved in the emergence of resistance to a given molecule; however, it is not involved in the spread of resistance to other molecules.

Therefore, contrary to current beliefs, supposed *abuse* (i.e., *quantity*) of antibiotics is *not* the key culprit for the strengthening of resistance in pathogens (spread of resistance to other molecules, i.e., more molecules becoming resisted as a result of a lower number of molecules having been introduced to the pathogens). Exposure of pathogens to new molecules is.

Based on this characteristic, even with current safeguards and programs aimed at reducing *quantity* of drug pressure on pathogens, it is evident that the situation will continue to exacerbate and there will come a point where we will not be able to handle it anymore because the molecule-dependency factor, which is the key factor involved in the development of resistance to multiple molecules, is currently being ignored and, worse, turns out to be an *exponential* rate factor, as seen [3]. Therefore, the opposite of the current understanding is true: because the pathogens’ rate of resistance emergence is *also* molecules-dependent and is greater than our rate of drugs production, a reduction in the use of antibiotics (*quantity*) can *not* and will *not* by itself lead to a subsidence in drug resistance, and the production of more antibiotics (including the re-use of old antibiotics in new cocktails) will only make matters worse. At this point, *radical* and *immediate* turnaround is *required*.

*Radical*, here, means *complete cessation* of the use of drugs or combination drugs as we currently understand them. Anything short of that will cost us and will continue to cost us dearly. This radical turnaround is *required* because, as seen, for every single molecule we introduce to pathogens to control or circumvent *n* existing cases of resistance, the pathogens return to us *n'* > n (new) cases of resistance. So, it is very evident that we are going bankrupt. Therefore, the turnaround needs to be *immediate* because the longer we hold onto our current practices, the tighter the situation gets.

Although those two (*radical* and *immediate*) conditions are tight ones in and of themselves, the implication of not meeting them is that resistance will continue to strengthen as we continue to lag farther and farther behind pathogens in terms of number of drugs available to combat resistance. Consequences will be unspeakable. The combination of such tight conditions certainly makes the situation so difficult to handle. At any rate, however, the current understanding needs in-depth rectification.

In fact, the only reason why we have been having a hard time controlling resistance is that there is a law at work (the first law of resistance) which we have unknowingly been violating. Abiding now by this law, drug conception as we currently understand it needs to undergo required changes and be brought into compliance with *Resistance Threshold* theory [6] in order to prevent resistance from arising in the first place. This will occur once we have understood that resistance is not specific to just pathogens but is a natural phenomenon displayed by all living organisms across the biological realm and is governed by specific laws.

Moving forward, the pharmaceutical industry has been blamed for not manufacturing antibiotics at a much greater rate. But it can now be seen that this much-disliked, often-attacked, low antibiotic production rate is so much beneficial to us, after all—for had it been higher, resistance would have been stronger. The opposite of the current situation (i.e., a higher drugs production rate, conceived according to our current understanding) would have strengthened pathogen resistance beyond its current level, making the crisis so much more dire than it currently is.

## Conclusion

Comparison of the resistance emergence rate in pathogens with the drug production rate by the pharmaceutical industry shows that we have already fallen behind in our quest to contain pathogen resistance. Furthermore, rates comparison reveals that it is impossible to win this competition in the future with the approach we are currently using. From the mathematics characterizing both the drug production and resistance emergence processes, it appears that a review of the current understanding on resistance control is needed. That molecules feed the strengthening of resistance in pathogens and that this resistance emerges at an exponential rate, much faster than we can produce drugs, has the immediate implication that there is a continuously widening gap between the strength of drug resistance and our ability to contain it with new molecules. The new approach, expected to overcome resistance once and for all, is, however, given by the first law of resistance applied to drug design [6].
